# Supplementary material for: Healthcare built environment and behavioural and physiological indicators of stress responses in autism spectrum disorder: Protocol for a mixed-methods systematic review
Source: PLoS One. 2026 Apr 20;21(4):e0347308. doi: 10.1371/journal.pone.0347308 (PMC13094996; doi:10.1371/journal.pone.0347308)
Supplement: S2 Appendix — (PDF) [file pone.0347308.s002.pdf]

S2 Appendix

Definition of "healthcare built environment" as used in this review.

First, the "built environment" for this review is defined as comprising the following (as was used by Lipson-Smith *et al.,* 2021):

a. ambient features (e.g., noise, air quality, odours, light, temperature);
b. architectural and landscape features (e.g., position and layout of the building, relationship between the building and its surroundings, dimensions of a room, placement of doors and windows, views and outdoor areas);
c. interior design features (e.g., furniture, artwork, signage, colours, equipment, and technology); and
d. maintenance and housekeeping (e.g., cleanliness, repair and upkeep of architectural and interior features).

Second, we define a "healthcare built environment" as the physical environment (comprising the features such as those listed above) of any primary, secondary and tertiary healthcare setting where inpatient or outpatient health services are delivered, including but not limited to general practice, hospitals, mental health services, etc. (excluding home care services).

Reference:

Lipson-Smith, R., Pflaumer, L., Elf, M., Blaschke, S.-M., Davis, A., White, M., Zeeman, H., & Bernhardt, J. (2021). Built environments for inpatient stroke rehabilitation services and care: a systematic literature review. *BMJ Open*, *11*(8), e050247. https://doi.org/10.1136/bmjopen-2021-050247
