# Supplementary material for: Healthcare built environment and behavioural and physiological indicators of stress responses in autism spectrum disorder: Protocol for a mixed-methods systematic review
Source: PLoS One. 2026 Apr 20;21(4):e0347308. doi: 10.1371/journal.pone.0347308 (PMC13094996; doi:10.1371/journal.pone.0347308)
Supplement: S3 Appendix — (PDF) [file pone.0347308.s003.pdf]

S3 Appendix

Complete search strategy used in the systematic review for Medline (Pubmed interface)

Key:
* = truncation
MeSH Terms = Medical Subject Headings

#	Search Queries
1	"autistic disorder"[MeSH Terms] OR "autism spectrum disorder"[MeSH Terms] OR "asperger syndrome"[MeSH Terms] OR "child development disorders, pervasive"[MeSH Terms] OR "developmental disabilities"[MeSH Terms] OR "autis*"[Title/Abstract] OR "ASD"[Title/Abstract] OR "asperg*"[Title/Abstract] OR "development* dis*"[Title/Abstract] OR "pervasive development*"[Title/Abstract] OR "pervasive child development*"[Title/Abstract] OR "PDD"[Title/Abstract] OR "PDD-NOS"[Title/Abstract] OR "PDDNOS"[Title/Abstract] OR "neurodevelopment* dis*"[Title/Abstract]
#2	"health facilities"[MeSH Terms] OR "health* setting*"[Title/Abstract] OR "health* facilit*"[Title/Abstract] OR "health* environment*"[Title/Abstract] OR "health* space*"[Title/Abstract] OR "health* building*"[Title/Abstract] OR "health* unit*"[Title/Abstract] OR "health* service*"[Title/Abstract] OR "health* cent*"[Title/Abstract] OR "medical facilit*"[Title/Abstract] OR "medical setting*"[Title/Abstract] OR "medical cent*"[Title/Abstract] OR "medical environment*"[Title/Abstract] OR "care cent*"[Title/Abstract] OR "care setting*"[Title/Abstract] OR "care environment*"[Title/Abstract] OR "care facilit*"[Title/Abstract] OR "care building*"[Title/Abstract] OR "care space*"[Title/Abstract] OR "care unit*"[Title/Abstract] OR "outpatient clinic*"[Title/Abstract] OR "inpatient clinic*"[Title/Abstract] OR "clinical setting*"[Title/Abstract] OR "clinical environment*"[Title/Abstract]
#3	"psychiatric unit*"[Title/Abstract] OR "health* institution*"[Title/Abstract] OR "psychiatric institution*"[Title/Abstract] OR "psychiatric facilit*"[Title/Abstract] OR "behavio* unit*"[Title/Abstract]
#4	"ambulatory *care"[Title/Abstract] OR "family practice*"[Title/Abstract] OR "general practice*"[Title/Abstract] OR "GP"[Title/Abstract]
#5	"matern* care"[Title/Abstract] OR "neonatal care"[Title/Abstract] OR "matern* cent*"[Title/Abstract] OR "birthing cent*"[Title/Abstract] OR "matern* clinic*"[Title/Abstract]
#6	"dental cent*"[Title/Abstract] OR "dental facilit*"[Title/Abstract] OR "dental setting*"[Title/Abstract] OR "dental clinic*"[Title/Abstract]
#7	"cancer cent*"[Title/Abstract] OR "oncolog* cent*"[Title/Abstract] OR "rehabilitation cent*"[Title/Abstract] OR "cancer facilit*"[Title/Abstract] OR "oncolog* facilit*"[Title/Abstract]
#8	"hospital*"[Title/Abstract] OR "emergency *care"[Title/Abstract] OR "emergency department*"[Title/Abstract] OR "emergency room*"[Title/Abstract] OR "emergency unit*"[Title/Abstract] OR "urgent care"[Title/Abstract] OR "intensive care"[Title/Abstract] OR "critical care"[Title/Abstract] OR "surgicent*"[Title/Abstract] OR "surg* cent*"[Title/Abstract] OR "surg* facilit*"[Title/Abstract] OR "surg*

	setting*"[Title/Abstract] OR "operati* room*"[Title/Abstract] OR "operati* theatre*"[Title/Abstract] OR "operati* suite*"[Title/Abstract] OR "surg* room*"[Title/Abstract] OR "surg* theatre*"[Title/Abstract] OR "surg* suite*"[Title/Abstract] OR "recovery room*"[Title/Abstract] OR "rehabilitation room*"[Title/Abstract] OR "preoperat* area*"[Title/Abstract] OR "preoperat* holding area*"[Title/Abstract] OR "perioperat* area*"[Title/Abstract]
#9	"pharmacy"[Title/Abstract] OR "pharmacies"[Title/Abstract] OR "resident* aged care"[Title/Abstract] OR "care home*"[Title/Abstract] OR "nursing home*"[Title/Abstract] OR "institutional* care"[Title/Abstract] OR "assisted living"[Title/Abstract] OR "aged care"[Title/Abstract] OR "long stay facilit*"[Title/Abstract] OR "long term facilit*"[Title/Abstract] OR "long term *care"[Title/Abstract] OR "medical home*"[Title/Abstract] OR "health* home*"[Title/Abstract]
#10	"waiting rooms"[MeSH Terms] OR "waiting room*"[Title/Abstract] OR "patients rooms"[MeSH Terms] OR "patient s room*"[Title/Abstract] OR "ward*"[Title/Abstract]
#11	#2 OR #3 OR #4 OR #5 OR #6 OR #7 OR #8 OR #9 OR #10
#12	"electroencephalography"[MeSH Terms] OR "neurofeedback"[MeSH Terms] OR "electro*encephal*"[Title/Abstract] OR "electrophysio*"[Title/Abstract] OR "Electrocerebral"[Title/Abstract] OR "EEG"[Title/Abstract] OR "qEEG"[Title/Abstract] OR "magnetoencephalography"[MeSH Terms] OR "magnet*encephalogr*"[Title] OR "MEG"[Title]
#13	"connectome"[MeSH Terms] OR "functional neuroimaging"[MeSH Terms] OR "Magnetic resonance imaging"[MeSH Terms:noexp] OR "connectom*"[Title/Abstract] OR "Magnetic resonance imaging"[Title/Abstract] OR "Neuroimaging"[Title/Abstract] OR "neuro imaging"[Title/Abstract] OR "MRI"[Title/Abstract] OR "fmri*"[Title/Abstract]
#14	"heart rate"[MeSH Terms] OR "heart rate"[Title/Abstract] OR "cardiac variab*"[Title/Abstract] OR "pulse varia*"[Title/Abstract] OR "HRV"[Title/Abstract]
#15	"galvanic skin response"[MeSH Terms] OR "electrodermal activity"[Title/Abstract] OR "EDA"[Title/Abstract] OR "electrodermal response"[Title/Abstract] OR "electrodermal*"[Title/Abstract] OR "skin conductance response"[Title/Abstract]
#16	"hydrocortisone"[MeSH Terms] OR "Cortisol"[Title/Abstract] OR "glucocorticoid*"[Title/Abstract] OR "corticosteroid*"[Title/Abstract] OR "steroid hormone*"[Title/Abstract] OR "leukocytes"[MeSH Terms] OR "leukocyte*"[Title/Abstract] OR "leucocyte*"[Title/Abstract] OR "granulocyte*"[Title/Abstract] OR "white blood cell*"[Title/Abstract] OR "WBC"[Title/Abstract] OR "WBCs"[Title/Abstract] OR "monocyte*"[Title/Abstract] OR "peripheral inflammatory *markers"[Title/Abstract] OR "circulating inflammatory *markers"[Title/Abstract] OR "interleukins"[MeSH Terms] OR "interleukin*"[Title/Abstract] OR "IL"[Title/Abstract] OR "ILs"[Title/Abstract]
#17	"eye movement measurements"[MeSH Terms] OR "eye movement*"[Title/Abstract] OR "eye-tracking"[Title/Abstract] OR "gaze tracking"[Title/Abstract]
#18	#12 OR #13 OR #14 OR #15 OR #16 OR #17
#19	"patient reported outcome measures"[MeSH Terms] OR "reported outcome*"[Title/Abstract] OR "reported experience measure*"[Title/Abstract]

	OR "prom*"[Title/Abstract] OR "prem*"[Title/Abstract] OR "PRO"[Title/Abstract] OR "PROs"[Title/Abstract]
#20	"patient satisfaction"[MeSH Terms] OR "Satisfaction"[Title/Abstract] OR "experience*"[Title/Abstract] OR "opinion*"[Title/Abstract] OR "perspective*"[Title/Abstract] OR "perception*"[Title/Abstract] OR "preference*"[Title/Abstract]
#21	"health care surveys"[MeSH Terms] OR "tool*"[Title/Abstract] OR "questionnaire*"[Title/Abstract] OR "survey*"[Title/Abstract] OR "scale*"[Title/Abstract] OR "instrument*"[Title/Abstract] OR "Index"[Title/Abstract] OR "indices"[Title/Abstract] OR "measure*"[Title/Abstract] OR "profile*"[Title/Abstract] OR "assess*"[Title/Abstract]
#22	#19 OR #20 OR #21
#23	"emotions"[MeSH Terms] OR "emotion*"[Title/Abstract] OR "feeling*"[Title/Abstract] OR "anxiety"[Title/Abstract] OR "stress"[Title/Abstract] OR "distress"[Title/Abstract] OR "behavior"[MeSH Terms] OR "behavio*"[Title/Abstract] OR "sensory processing"[Title/Abstract] OR "sensory"[Title/Abstract] OR "mood"[Title/Abstract] OR "daily functioning"[Title/Abstract]
#24	#22 AND #23
#25	#18 OR #24
#26	#1 AND #11 AND #25
#27	(review[pt] OR systematic review[pt] OR review[ti])
#28	#26 NOT #27
#29	limit #28 to yr="2014-2025"
