# Supplementary material for: Healthcare built environment and behavioural and physiological indicators of stress responses in autism spectrum disorder: Protocol for a mixed-methods systematic review
Source: PLoS One. 2026 Apr 20;21(4):e0347308. doi: 10.1371/journal.pone.0347308 (PMC13094996; doi:10.1371/journal.pone.0347308)
Supplement: S4 Appendix — (PDF) [file pone.0347308.s004.pdf]

S4 Appendix

Variables collected in pre-established data extraction template.

Construct	Variables
(A) Study information	• First author's last name, publication year<br>• Journal name<br>• Geographical region in which the study was conducted<br>• Duration of follow-up or study period<br>• Aims and/or purpose of the study
(B) Research design and methodology	• Study design<br>• Duration<br>• Follow-up<br>• Data collection<br>• Statistical analysis
(C) Participant, patient characteristics	• Sample size<br>• Eligibility criteria<br>• Gender composition<br>• Mean or median age<br>• Ethnicity<br>• Socio-economic status (if available)
(D) Clinical characteristics (if available)	• Comorbidities: medical classification topography code, collection method, measure of association[1]<br>• Baseline clinical parameters<br>• Body mass index<br>• Prescribed medications<br>• Sensory processing characeristics
(E) HC setting	• Study setting<br>• Healthcare typology<br>• Co-Interventions or exposure HC-BE feature
(F) Healthcare built environment intervention/exposure	• Device(s), technology and/or BE feature information<br>• Technical specifications<br>• Duration of treatment/observation period<br>• Timing, delivery<br>• Economic information
(G) improved/modified environmental factor (if applicable)	• Parameter type within categories (i.e., pollutants)<br>• Sources<br>• Exposure levels<br>• Method(s) of measurement<br>• Equipment<br>• Measurements: dose, duration, intensity, frequency
(H) Physiological biomarkers	• Biomarker types grouped by system<br>• Circulating biomarkers (laboratory assay methods, Cut-offs, midpoints to report up- or down-regulation, concentration level (pre- and post-study/intervention)

	• Neurophysiology. EEG/MEG technique/paradigm, recording system/duration, used channels.<br>• Neuroimaging. fMRI methods/paradigms, description of the task design (if applicable for task-based fMRI), documentation of the ROI selection process, modality of stimuli, quality control steps.[1]<br>• Autonomic nervous system. HRV measuring methods, HRV measure derived, duration of HRV-sampling (long- [approx. 24h], short-[approx. 5 min], and ultra-short-ter,[< 5 min]), sensor type used.<br>• EDA methods, EDA signal processing (filtering- noise removal, cleaning- exclusion of movement artifacts), and normalization methods.<br>• Behavioural. Type of eye tracking technology, dynamic mobility task, eye tracking measures used (fixation durations, gaze durations, fixation locations, and/or saccadic movements).
(I) Behavioural indicators	• PROMs/PREMs name[2]<br>• PROMs/PREMs description<br>• Domains PROM/PREM is measuring
(J) Outcomes (where applicable for each biomarker/indicator)	• Analysis models<br>• Effect estimates<br>• Adjusted potential confounders<br>• Statistical thresholds<br>• Time point<br>• Measurement instruments
(K) Results	• Key findings

EDA, electrodermal activity markers; EEG, electroencephalography; fMRI, functional magnetic resonance imaging; HC-BE, Healthcare-Built Environment; HRV, Heart Rate Variability; MEG, magnetoencephalography; PREMs, Patient-Reported Experience Measures; PROMs, Patient-Reported Outcome Measures; ROI, Regions of Interests.

[1]Data extraction in accordance with published fMRI reporting guidelines: Poldrack RA, Fletcher PC, Henson RN, et al. Guidelines for reporting an fMRI study. Neuroimage 2008; 40: 409–414.

[2] We use Patient-Reported Outcome Measures (PROMs) and Patient-Reported Experience Measures (PREMs) in the table for simplicity. However, we refer to self-, caregiver-, HC professionals-reported data.
