# Supplementary material for: Healthcare built environment and behavioural and physiological indicators of stress responses in autism spectrum disorder: Protocol for a mixed-methods systematic review
Source: PLoS One. 2026 Apr 20;21(4):e0347308. doi: 10.1371/journal.pone.0347308 (PMC13094996; doi:10.1371/journal.pone.0347308)
Supplement: Attachment — Submitted filename: Response to Reviewers.pdf [file pone.0347308.s006.pdf]

RESPONSE TO REVIEWER #1 (PONE-D-25-32161)

Point 1: The objective as last paragraph in Introduction section will give clarity to the readers.

Response 1: We thank the reviewer for this comment. We have added a more concise objective paragraph at the end of the Introduction to provide greater clarity regarding the aims of the review. The revised text now reads: "This systematic review aims to explore whether the exposure to specific HC-BE features can alter behavioural and physiological indicators of anxiety, stress, and emotion in ASD, which is associated with atypical sensory processing, integrating both qualitative and quantitative evidence to identify effective design strategies."

Point 2: It will be interesting to see how the qualitative and quantitative findings would be integrated.

Response 2: We thank the reviewer for this insightful comment. We agree that integrating qualitative and quantitative findings is crucial in our systematic review. This approach was already described in the manuscript, Methods/Data Synthesis section: "If there is a combination of quantitative and qualitative studies, a results-based convergent synthesis design is planned, with independent syntheses of quantitative and qualitative data, followed by integration of evidence derived from both syntheses in a third synthesis in accordance with Joanna Briggs Institute methodology for conducting a mixed-methods systematic review [40]." We also believe that our planned approach will provide a clear and rigorous framework for combining multiple types of evidence.

Point 3: 10 Biomarkers have been used. The philosophical rationale behind why eye-tracking and salivary cortisol were prioritized over others would strengthen methodological foundation.

Response 3: We thank the reviewer for this comment. We would like to clarify that in our manuscript all ten non-invasive biomarkers listed in Table 2 are considered equally; no emphasis or prioritization is placed on eye-tracking or salivary cortisol over the other biomarkers. In the manuscript, we clearly stated that these ten biomarkers were selected because they showed the most consistent results across systematic reviews, which follow rigorous methodology. This selection approach ensures that all biomarkers are included on an equal basis. We have additionally clarified this point in the Outcomes section to avoid any potential misunderstanding regarding the selection of biomarkers with the following sentence: "All ten non-invasive biomarkers were included based on their consistent results in prior systematic reviews, ensuring a rigorous and unbiased selection."

Point 4: Use of terminologies should be consistent eg. HC – BE.

Response 4: We thank the reviewer for this comment. We have carefully reviewed the manuscript and ensured that the terminology, particularly "HC-BE", is now used consistently throughout the text, tables, and figures.

Point 5: Acronyms (e.g., EEG, MEG) appears in tables without being defined in the main text.

Response 5: We thank the reviewer for this comment. We have double-checked the main text and tables to ensure that each acronym matches its first definition and that the abbreviations are used consistently throughout the manuscript.

Point 6: Grammatical and typographical errors have to be checked for.

Response 6: We thank the reviewer for pointing this out. We have carefully reviewed the manuscript for grammatical and typographical errors and corrected them to ensure clarity and readability throughout the text and tables.

Point 7: A summary Review Literature Review table bringing out the gaps would add value.

Response 7: We thank the reviewer for this thoughtful suggestion. We fully agree that a summary table of the existing literature can often add value in a manuscript. In this case, however, we are presenting a protocol for a mixed-methods systematic review. The primary aim of the protocol is to outline the methodology that will be applied to systematically identify, appraise, and synthesize the existing evidence. Preparing a literature review table at this stage, before conducting the systematic search, screening, and appraisal steps, could introduce bias regarding which studies, gaps, populations, or outcomes are highlighted. Since the systematic review itself will provide a rigorous and transparent synthesis of the literature, including such a table at the protocol stage would risk being redundant and potentially misleading. We have carefully considered the reviewer's suggestion but have chosen not to include a review summary table in the current manuscript to preserve methodological rigor and avoid providing readers with potentially biased information. Instead, the forthcoming mixed-methods systematic review will incorporate a structured data extraction process, as outlined in the Data Extraction section, and will present tabular summaries of findings where appropriate; the most suitable synthesis approach will be determined after data extraction and quality assessment, as described in the Data Synthesis section of the manuscript.

RESPONSE TO REVIEWER #2 (PONE-D-25-32161)

Point 1: Synthesize the findings of multiple research studies on this topic, using a rigorous and transparent methodology . Include more studies on this work.

Response 1: We thank the reviewer for this comment and fully agree that synthesizing findings from multiple studies using a rigorous and transparent methodology is essential. We would like to clarify, however, that the present manuscript is a protocol for a mixed-methods systematic review. Its purpose is to describe in detail the methodology that will be applied to systematically identify, appraise, and synthesise the relevant studies following the screening process. The actual synthesis of findings and inclusion of all eligible studies will be conducted in the subsequent systematic review, in accordance with the procedures outlined in this protocol.

Point 2: Avoid using we, I etc in paragraphs eg:-" We will search for peer-reviewed articles in PubMed", Systematic Reviews and MetaAnalyses guidelines will be followed , "Risk of bias will be" etc……

Response 2: We thank the reviewer for this helpful comment. We recognize that excessive use of first-person pronouns (e.g., “we will…”) can detract from a neutral and objective tone in scientific writing. While PLOS ONE permits the use of first-person pronouns, we agree that greater neutrality is preferable when describing the methodology of the protocol. To address this, we have revised the manuscript to limit first-person usage specifically in sections outlining methodological procedures (e.g., changed “We will search for peer-reviewed articles in PubMed” to “Peer-reviewed articles will be searched in PubMed”). This adjustment ensures that the methodological descriptions remain objective and rigorous, while retaining clarity and readability in other sections of the manuscript.

Point 3: You have generalised the Built Environment studies and physiological indicators, elaborate the work on each environment given in literature and cite the reference.

Response 3: We appreciate the reviewer’s comment. In the Introduction, we have added specific details on which BE features were studied in prior systematic reviews and, where available, their links to corresponding physiological and behavioural biomarkers. We would like to clarify, however, that the primary aim of this protocol is to systematically link HC-BE-specific details with the most consistent biomarkers identified in previous studies. To select the appropriate biomarkers, we consulted systematic reviews of clinical data. The protocol outlines how the review will extract and synthesise HC-BE-specific information associated with physiological and behavioural biomarkers. For each type of HC-BE (e.g., hospital wards, outpatient clinics, sensory rooms), the review will systematically record details on HC-BE features and their evaluation alongside the corresponding biomarkers. This process is fully described in the Data Extraction and Data Synthesis sections of the manuscript.

Point 4: I find more redundancy in the manuscript.

Response 4: We acknowledge the reviewer’s observation regarding perceived redundancy. To address this, we carefully reviewed the entire manuscript and implemented several revisions to enhance conciseness and readability:

1. Introduction and Discussion: Merged sentences that previously described HC-BE features, physiological and behavioural biomarkers, and their interrelations, using more concise and streamlined phrasing.
2. Methodology: Replaced repeated restatements of the study aim and methodological details with references to the appropriate sections, thereby reducing repetition while maintaining clarity.
3. Tables: Reviewed all tables and corresponding main text passages, removing duplicated explanations to avoid redundancy and improve readability.

These changes ensure the manuscript presents the scientific content in a coherent, logically structured manner without unnecessary repetition.

Point 5: You have just specified the name of biomarkers used in the study , Need to explain how it is used , outcome etc and should have complete information about each biomarker.

Response 5: We appreciate the reviewer’s comment. In the revised manuscript, we have expanded the Outcomes section to provide detailed information about each biomarker.

Specifically, we now describe how each biomarker is assessed and how outcomes will be measured (e.g., baseline levels, pre–post intervention changes, or cut-off thresholds where applicable). This detailed information informs the planning of data synthesis for quantitative outcomes, and we have added a corresponding description in the Data Synthesis section. These revisions ensure that the Outcomes and Data Synthesis sections are fully aligned with PRISMA guidelines and enhance transparency for future replication.

Point 6: Table 3 is repeated content from the paragraph. What the reader can infer from the table?

Response 6: We thank the reviewer for highlighting this point. After careful consideration, we agree with the reviewer that Table 3 does not present additional, unique information beyond the main text. We have now provided a detailed and coherent description of the biomarkers and behavioural measures, and have applied the same approach for Table 2. Therefore, to avoid redundancy and maintain readability, we have decided not to include Tables 2 and 3.

Point 7: Which author extract the data? Are you reviewing a work done in literature study "Data extraction will be conducted by one author (BW)"-Page 11.

Response 7: In this systematic review, data extraction will be conducted by one author (BW) and then verified using a crowdsourcing approach. We have now explicitly specified the members of the review team who will verify the data: Daryia Palitika (DP) and Mar Garcia Rodriguez (MGR). The crowdsourcing approach is widely accepted in systematic reviews when clearly reported, as it ensures accuracy and consistency. This method also allows the inclusion of additional members, identified via Cochrane Task Exchange, to assist with non-English articles, supporting a rigorous and reliable synthesis of the literature.

Point 8: Study Selection:- Need to know how to synthesize the study not how you use reference manager.

Response 8: We thank the reviewer for the comment. The procedures for synthesizing the included studies are already detailed in the manuscript. In the Data Extraction section, we describe the variables to be collected and how data will be verified. In the Data Synthesis section, we explain the planned integration of quantitative and qualitative findings using a results-based convergent synthesis design, and the potential use of random-effects meta-analysis for quantitative outcomes. The information provided in the Study Selection section follows PRISMA guidelines, which require a clear description of the process used to identify and screen studies. The mention of the reference manager refers only to organizational purposes and is not part of the synthesis process.

Point 9: Expand the abbreviation at the beginning of the passage followed by its short form in rest of the paragraph.

Response 9: We acknowledge the reviewer's observation. We have now revised the manuscript to ensure that all abbreviations are expanded at first mention and consistently abbreviated thereafter.
