# Supplementary material for: Healthcare built environment and behavioural and physiological indicators of stress responses in autism spectrum disorder: Protocol for a mixed-methods systematic review
Source: PLoS One. 2026 Apr 20;21(4):e0347308. doi: 10.1371/journal.pone.0347308 (PMC13094996; doi:10.1371/journal.pone.0347308)
Supplement: Attachment — Submitted filename: Response_to_Reviewers_auresp_2.pdf [file pone.0347308.s007.pdf]

RESPONSE TO REVIEWER #3

Comment 1: Major part of the discussion is focussed on built environment design and briefly on the impact of the behaviour of the children of autism. Further the language used in discussion is in future tense .No conclusive results shown.
Limitations of the study not mentioned. No future directions given.
No dedicated section on Results. There is no mention of the documentation of the research process in log document.
There is no mention of how many research papers were reviewed and of which how many were potentially relevant articles were retrieved. There is no summary of person characteristics of behaviour to understand the impact of built environment.

Response 1: We would like to clarify that the submitted manuscript is a protocol for a mixed-methods systematic review, not a completed review reporting findings. The manuscript has been developed following PRISMA-Protocols guidelines. As a protocol, several elements noted by the reviewer, such as the number of included studies, participant characteristics, and results, are not expected at this stage. The use of future tense throughout reflects the planned methodology.

The review aims to examine how features of the HC-BE influence behavioural and physiological indicators of stress responses in autistic individuals. Accordingly, the Discussion presents relevant aspects of the HC-BE on this topic, their theoretical and historical context, and the anticipated implications for this field, while acknowledging that the actual impact on outcomes will only be determined once the review is conducted.

RESPONSE TO REVIEWER #4

Major concerns

Point 1: Page 4: "Interestingly, a state of hyperarousal evaluated through salivary cortisol (CT) in autism demonstrates correlated associations with heightened indices of behavioural anxiety/stress response [21]."
I think that this sentence is confusing and/or misleading. In the article cited by the authors, "heightened repetitive language (subscale of RBQ) was positively correlated with the Total Anx-DOS Score (rs (22) = 0.452, p = .027)", a behavioral index of anxiety. "There were no significant correlations between Total Anx-DOS score and subscale scores from the SRS-2 or RBQ." Meaning that the association mentioned above was only valid for one of the two neurogenetic disorders included in the study. No association was found in the study between Anx-DOS and salivary cortisol. So in all I think this sentence should be removed or modified to better reflect what was found or not in the original study.
Crawford, H., Oliver, C., Groves, L., Bradley, L., Smith, K., Hogan, A., Renshaw, D., Waite, J., & Roberts, J. (2023). Behavioural and physiological indicators of anxiety reflect shared and distinct profiles across individuals with neurogenetic syndromes. Psychiatry Research, 326, 115278. https://doi.org/10.1016/j.psychres.2023.115278

Response 1: We thank the reviewer for this important clarification and agree that the original wording was potentially misleading. In response, we have revised the sentence to remove references to hyperarousal and direct behavioural–physiological coupling, and to more accurately reflect the syndrome-specific and heterogeneous nature of the findings. The revised

text now emphasizes altered basal physiological arousal in neurogenetic syndromes linked to autistic phenotypes without overstating associations with behavioural anxiety measures.

Point 2: Page 7: "Both inpatient and outpatient HC-BE will be included, however, those purposively designed for autistic users will not be considered"

I don't understand this choice. Why not treat these studies as a subgroup of data to compare with environments that have not been purposively designed for autistic users? I think this could lead to very interesting results on what specific aspects of BE affect autistic persons' preferences and well-being? Please provide a rationale for this choice.

Response 2: Our decision to exclude care environments purposively designed for autistic users is grounded in the conceptual aims of our review. Since most autistic individuals receive care in standard HC facilities rather than specialist units, our focus is on understanding their real-world experiences within care environments that were not designed with autism in mind.

It is important to clarify that, to our best knowledge, national or international HC design guidance—including comprehensive frameworks such as the UK Health Building Notes—does not include autism-specific accessibility requirements. This gap is also reflected in HC design guidance reviewed in other research projects across multiple countries. Consequently, any HC facility not explicitly created as an autism-specific unit cannot be considered "purpose-built" for autistic users. Mainstream HC settings such as general hospitals, maternity units, dental clinics, primary care facilities, and most psychiatric wards are designed for the general population, with the needs of autistic people absent from the design briefs that inform these environments.

Including specialist autism-designed settings would therefore introduce a fundamentally different design context and fall outside our aim of identifying BE features that affect autistic individuals in the standard HC facilities they encounter across their lifespan.

Point 3: Page 8: "EEG measures cortical electrical activity across frequency bands (e.g., alpha, beta, theta), while MEG detects rapid changes in brain responses to sensory stimuli."

I don't think that this sentence reflects accurately what EEG and MEG can tell us about brain activity. The main difference between the two methods is in the nature of the signal: electrical activity measured through captors placed on the scalp for EEG, reflecting the neurons' action potentials; subtle changes in the magnetic field for MEG, also reflecting the neuron's action potentials. I don't think that these two measures differ in how they capture spontaneous or evoked activity. They both can do that. Please correct this sentence accordingly.

Response 3: We appreciate the reviewer's careful reading and agree that our original sentence did not accurately characterise the distinction between EEG and MEG. We have revised the sentence in the manuscript to more accurately reflect the nature of these neurophysiological measures, as follows:

"EEG and MEG both measure neural activity arising from synchronous postsynaptic currents, with EEG detecting electrical potentials at the scalp and MEG detecting the associated magnetic fields; both methods can record spontaneous and evoked activity."

Point 4: Page 4: "impact neural pathways and structures of emotional processing [19]."

The notion of neural pathways in my opinion reflect physiological data pertaining to connections in the brain, such as Diffusion Tensor Imaging. Is this what is intended by the authors? Maybe simply replace "pathways" by "activity" to reflect that you aggregated data pertaining to structural and functional imaging?

Response 4: We thank the reviewer for this suggestion. To avoid potential misinterpretation that the term "neural pathways" refers only to anatomical connections, we have replaced it with "neural activity". The revised sentence now reads:

"impact neural activity and structures of emotional processing [19]."

Point 5: Discussion section.

Most of the paragraphs from this section seem like they could also be placed in the Introduction section, except for the final paragraph. I don't know if it's a problem or not. I don't really know what is expected in the Discussion section of an article presenting a review research protocol, since no results exist yet... Please refer to the Editor decision regarding this concern.

Response 5:

We thank the reviewer for this comment. We note that in a systematic review protocol, the Discussion section often overlaps with the Introduction, as there are no results yet to interpret. According to PRISMA-P (Item 15) guidance, the Discussion in a protocol (an optional and forward-looking section) is primarily intended to anticipated contributions and significance, and potential implications of the planned review. Our Discussion has been structured accordingly to: (i) emphasise the current evidence to highlight the expected contributions and the need for multi-method assessment of behavioural and physiological responses, (ii) provide historical and theoretical context relevant to the field, essential for contextualising the review, and (iii) outline the expected value and implications of the planned review (for research, practice, and policy). We think this structure is consistent with PRISMA-P recommendations for review protocols.

According to PRISMA-P (Items 6–8), the Introduction provides the key background and rationale for the review, identifies the main gaps in existing knowledge, clearly defines the systematic review question (framed in PI/ECO(S) terms), and provides brief context for eligibility criteria.

Reference: Moher D, et al. PRISMA-P 2015 statement. Systematic Reviews. 2015;4:1.

Minor concerns

Point 1: Abstract: "A growing body of systematic studies links autism-friendly BEs with positive care experiences, yet substantial gaps remain in understanding the effects on behavioural and physiological responses of emotion"

I think the formulation “the effects on behavioural and physiological aspects of emotional responses” would be more exact. “Responses of emotion” is not really a valid statement in my opinion.

Response 1: we thank the reviewer for this comment. We have revised the wording to improve conceptual clarity, replacing “responses of emotion” with “behavioural and physiological aspects of emotional responses.”

Point 2: Page 3: “The HC built environment (HC-BE) affects vulnerable individuals and influences patients' perceptions and sense of agency in ways that may not impact those who self-identify as healthy [7] and this might not be an exception for autistic people as explored in this review”

I find this sentence very complex to understand. Maybe use this formulation instead?: “While the HC built environment (HC-BE) may not impact much those who self-identify as healthy [7], it could affect vulnerable individuals and influence patients' perceptions and sense of agency, and this might not be an exception for autistic people as explored in this review”.

Response 2: We thank the reviewer for this helpful suggestion. Upon review, we agree that the original sentence may have been overly complex. We have revised the wording to improve readability while keeping the original meaning. The revised sentence now reads:

“While the HC built environment (HC-BE) may have limited impact on those who self-identify as healthy [7], it can affect vulnerable individuals and influence patients’ perceptions and sense of agency, and this might not be an exception for autistic people as explored in this review.”

Point 3: Page 3: “This effect may occur across intimate, private, and public HC-BE settings”

I understand what private and public HC-BE settings could be. But what could be an intimate HC-BE setting?

Response 3: We thank the reviewer for raising this point. In the HC-BE literature and field, “intimate” settings refer to small-scale, high-proximity clinical spaces where patients experience close interpersonal interaction and heightened vulnerability. This term reflects established usage within the field and captures a dimension of HC space that is particularly relevant when considering stress responses in autistic individuals.

Point 4: Page 5: Please explain the PI/ECOS acronym at first use.

Page 8: Same for Salivary CT

Response 4: The PI/ECOS acronym has now been introduced and defined at first use in the manuscript. Regarding salivary CT, the acronym for salivary cortisol was already defined on page 4.

Point 5: Page 8: typo. There is a missing bullet point in front of the “behavioral” section.

Response 5: We have carefully checked the manuscript and could not identify a missing bullet point before the "Behavioural" section. We have ensured that all bullet points are consistent and correctly formatted throughout the text.

Point 6: Page 11: "(c) participant, patient characteristics: sample size, participant characteristics, eligibility criteria ».

« Participant characteristics » is repeated twice.

Response 6: We thank the reviewer for noticing this repetition. We have revised the text to remove the duplicated phrase.

Point 7: Page 14 : « In this case, findings will be synthesised narratively, with with attention to baseline".

Please remove one "with".

Response 7: We have removed the duplicated "with" in the manuscript.

Point 8: Page 15: "This would negate the need for clinicians to visit the person requiring care at a specialised autism institution or for autistic individuals to visit the hospitals from the institutions, and therefore the institution overseeing their hospitalization"

I don't understand this sentence. Could you please rephrase it?

Response 8: We thank the reviewer for highlighting this sentence. We have revised it for clarity, replacing "negate" with "remove" and simplifying the structure. The revised sentence now reads:

"This would remove the need for clinicians to travel to specialised autism institutions, or for autistic individuals to visit hospitals via such institutions, thereby reducing the involvement of the institutions overseeing their hospitalisation."

RESPONSE TO REVIEWER #5

Point 1: Data availability statement should be part of the manuscript.

Response 1: We thank the reviewer for this comment. We have introduced a new Data Availability Statement section, which can be found on page 16 of the revised manuscript.

Point 2: The need for this review stems from the fact that autistic individuals experience anxiety (which includes stress) as well as sensory processing. What this protocol has failed to account for is that there are degrees of anxiety as well as degrees of sensory processing. The results of this review should evaluate the results for autistic individuals diagnosed with an anxiety disorder vs. autistic individuals not diagnosed with an anxiety disorder (that most certainly experience a certain level of anxiety in HC settings), as well as the results for autistic individuals that have received therapy for a sensory processing disorder (since diagnosis is not recognized in DSM-5)

vs. autistic individuals that have not received therapy for a sensory processing disorder (that might experience a certain level of sensory issues in HC settings). This info may not be available in all studies, but it is bound to be present in quite a few. Other planned subgroup analyses (e.g., by age, co-occurring conditions, or cognitive level) should also be included in the protocol to address heterogeneity in the ASD population.

Response 2: We thank the reviewer for this helpful comment. We would like to clarify that anxiety disorders were already considered within the category of comorbidities in our data extraction plan (Section 2.5, subsection d; page 12). To improve the focus on it, we have now explicitly specified anxiety disorders under the "comorbidities" variable ("clinical characteristics" subsection) in the manuscript. We agree that sensory processing differences are a core feature in our study. Consequently, we have clarified under the same "clinical characteristics" variable that sensory processing characteristics and related interventions (e.g., receipt of therapy targeting sensory processing challenges), where reported, will also be extracted. These clarifications were made to ensure that heterogeneity in anxiety and sensory processing is captured more explicitly.

Furthermore, we have specified that planned subgroup analyses will include anxiety disorder diagnosis versus no formal diagnosis, receipt of therapy for sensory processing challenges versus none, as well as age and co-occurring conditions, to account for heterogeneity within the autistic individuals. To reflect this, a sentence describing the planned subgroup analyses has been added to the Data Synthesis section (Section 2.7; page 14).

The Supporting Information (S4 Appendix) has been updated accordingly.

Point 3: Somewhat related to point 2 above, since the underpinning of this review is anxiety and sensory processing, it is important to mention in the introduction what % of autistic individuals are diagnosed with an anxiety disorder as well as what % typically undergo therapy for sensory processing. This should be framed in the context that the rest of the autistic individuals do experience anxiety, which may not typically reach clinical levels (hence the lack of diagnosis) - and a similar point for sensory processing.

Response 3: We thank the reviewer for this comment. To address this point, we have revised the Introduction to explicitly report the prevalence of diagnosed anxiety disorders in autistic individuals based on the meta-analysis by Micai et al. [5], already cited in the manuscript. To clarify that anxiety symptoms are commonly experienced, and that sensory processing differences are present even when formal diagnoses are absent, we added a new sentence supported by two additional references. These changes aim to contextualise the dimensional nature of anxiety and sensory processing that underpins the rationale for this review. We note that while sensory processing differences are well-established and widely reported, there is insufficient evidence to provide a reliable estimate of the percentage of autistic individuals who receive therapy specifically targeting sensory processing.

<u>Text added:</u>

"Approximately 35% of autistic individuals are diagnosed with an anxiety disorder; [5] however, many experience elevated anxiety symptoms that may not meet formal diagnostic thresholds, [6] as well as sensory processing differences [7]."

<u>References added:</u>

6. Torices Callejo L, Herrero L, Perez Nieto MA. Anxiety and autistic traits in adults: a systematic review and meta-analysis. Front Psychol. 2025;16:1680267. Epub 2025-10-29. doi: 10.3389/fpsyg.2025.1680267. PubMed PMID: 41235276; PubMed Central PMCID: PMCPMC12605409.

7. Chen Y, Xi Z, Saunders R, Simmons D, Totsika V, Mandy W. A systematic review and meta-analysis of the relationship between sensory processing differences and internalising/externalising problems in autism. Clin Psychol Rev. 2024;114:102516. Epub 2024-10-29. doi: 10.1016/j.cpr.2024.102516. PubMed PMID: 39515075.

Point 4: It is admirable that the authors chose to use identity first in this protocol. For consistency, in Table 1, please rephrase "patients with ASD" which is the same as "patients with autism" with "autistic patients" or "patients diagnosed with ASD." Similarly, in section 3. Discussion, please replace "those with physical disabilities" with physically disabled individuals."

Response 4: We have updated Table 1 to replace “patients with ASD” with “autistic patients” to ensure consistency with the identity-first language used throughout the manuscript and revised the Discussion section to replace “those with physical disabilities” with “physically disabled individuals.”

Point 5: People that are meant to ultimately benefit from the results of these study (autistic individuals, caregivers of autistic individuals) should be included in some manner in the dissemination of the results - to be included in Section 2.8.

Response 5: We thank the reviewer for this important comment. We have revised Section 2.8 to explicitly include autistic individuals and their caregivers as key stakeholders in the dissemination of the results, as follows: “Accessible summaries will be shared with autistic individuals, their caregivers, and relevant advocacy groups to ensure the research reaches those who may benefit most.”
