# Supplementary material for: Healthcare built environment and behavioural and physiological indicators of stress responses in autism spectrum disorder: Protocol for a mixed-methods systematic review
Source: PLoS One. 2026 Apr 20;21(4):e0347308. doi: 10.1371/journal.pone.0347308 (PMC13094996; doi:10.1371/journal.pone.0347308)
Supplement: Attachment — Submitted filename: RESPONSE TO REVIEWERS_PONE-D-25.docx [file pone.0347308.s008.docx]

**RESPONSE TO REVIEWER #3**

**Comment 1:** Reviewer #3: Corrections in the manuscript as required by the reviewers have been made. However I would suggest a small correction in page 9 the acronyms used for autonomic measures HRV and EDA to be written in expanded form.

There is no information on the limitations of this study.

**Response 1:** We truly appreciate your careful reading and constructive feedback in our protocol manuscript. We would like to kindly note that the acronyms for the two terms Heart rate variability (HRV) and electrodermal activity markers (EDA) were already introduced on page 8. For this reason, we continued using the acronyms on page 9 to maintain consistency and avoid repetition.

**RESPONSE TO REVIEWER #4**

Reviewer #4: I think that the authors did a great job in answering my concerns. I just have two minor concerns left, listed below, more as ideas to improve the manuscript.

**Comment 1:** I think that the authors’ response to my initial major concern n°2 is really great. Maybe the authors should consider including their response to my comment in the main text of the manuscript? Right after the sentence “Both inpatient and outpatient HC-BE will be included, however, those purposively designed for autistic users will not be considered”, page 7? I copy pasted their answer below.

"Our decision to exclude care environments purposively designed for autistic users is grounded in the conceptual aims of our review. Since most autistic individuals receive care in standard HC facilities rather than specialist units, our focus is on understanding their real world experiences within care environments that were not designed with autism in mind. It is important to clarify that, to our best knowledge, national or international HC design guidance—including comprehensive frameworks such as the UK Health Building Notes—does not include autism specific accessibility requirements. This gap is also reflected in HC design guidance reviewed in other research projects across multiple countries. Consequently, any HC facility not explicitly created as an autism specific unit cannot be considered “purpose built” for autistic users. Mainstream HC settings such as general hospitals, maternity units, dental clinics, primary care facilities, and most psychiatric wards are designed for the general population, with the needs of autistic people absent from the design briefs that inform these environments. Including specialist autism designed settings would therefore introduce a fundamentally different design context and fall outside our aim of identifying BE features that affect autistic individuals in the standard HC facilities they encounter across their lifespan."

**Response 1:** We sincerely thank the reviewer for this excellent suggestion. We are very pleased that our previous response addressed your initial major concern. We have now incorporated the clarified explanation directly into the main text of the manuscript, immediately after the sentence: “Both inpatient and outpatient HC-BE will be included, however, those purposively designed for autistic users will not be considered” (page 7). We slightly shortened it to ensure clarity and conciseness within the flow of the manuscript:

“The decision to exclude care environments purposely designed for autistic users is grounded in the conceptual aims of this review. Since most autistic individuals receive care in standard HC facilities rather than specialist units, our focus is on understanding their real-world experiences within care environments that were not designed with autism in mind. To our best knowledge, national and international HC design guidelines do not include autism specific accessibility requirements, and this gap is reflected across design guidance reviewed in other research projects across multiple countries. As a result, most HC facilities—including general hospitals, maternity services, dental clinics, primary care, and most psychiatric wards—are not purpose‑built for autistic users. Including specialist autism designed settings would therefore introduce a fundamentally different design context and fall outside the scope of identifying BE features that affect autistic individuals in standard HC settings they encounter across their lifespan.”

**Comment 2:** Regarding my initial minor concern n°3, please provide a short definition or some examples of “intimate, private, and public HC-BE settings” in the main text of the manuscript, page 3, in the Introduction section.

**Response 2:** We thank the reviewer for this suggestion. We have carefully considered adding brief definitions of “intimate, private, and public HC‑BE settings” into the Introduction. However, the reference we cite in this section already provides the conceptual grounding needed for these terms.

Because our Introduction is structured to present the overarching theoretical framing rather than detailed typologies of HC‑BE spaces, inserting definitions for each category would disrupt the narrative flow and shift focus away from the core conceptual argument. For this reason, we have opted not to add full descriptions of all three terms directly into the main text.

Nonetheless, to support clarity, we have included a concise parenthetical clarification for “intimate settings” without lengthening the text or interrupting its trajectory: “(i.e., small, enclosed spaces with heightened vulnerability)”.

**RESPONSE TO REVIEWER #5**

**Comment 1:** The authors have addressed all five points raised in my previous review in a thorough and satisfactory manner. Specifically: (1) a Data Availability Statement has been added to the manuscript body; (2) subgroup analyses stratifying by anxiety disorder diagnosis, receipt of sensory processing therapy, age, and co-occurring conditions have been explicitly incorporated into the manuscript; (3) the Introduction now includes the prevalence of diagnosed anxiety disorders (~35%) and acknowledges that many autistic individuals experience sub-threshold anxiety symptoms as well as sensory processing differences, supported by two new references; (4) identity-first language has been applied consistently, including in Table 1 and the Discussion; and (5) Section 2.8 now explicitly names autistic individuals and their caregivers as stakeholders in dissemination. I have no further concerns. The protocol is well-structured, methodologically sound, and appropriately follows PRISMA-P guidelines. I recommend acceptance.

**Response 1:** Thank you very much for your positive assessment and supportive feedback. We are grateful for your careful review and are pleased that you find the protocol well‑structured, methodologically sound, and aligned with PRISMA‑P guidelines.
