# Supplementary material for: Healthcare built environment and behavioural and physiological indicators of stress responses in autism spectrum disorder: Protocol for a mixed-methods systematic review
Source: PLoS One. 2026 Apr 20;21(4):e0347308. doi: 10.1371/journal.pone.0347308 (PMC13094996; doi:10.1371/journal.pone.0347308)
Supplement: Attachment — Submitted filename: Reviewer Comments-PONE-D-25-32161.docx [file pone.0347308.s005.docx]

**Reviewer Comments**

**Manuscript Title:-“Healthcare built environment and behavioural and physiological indicators of stress responses in autism spectrum disorder: Protocol for a mixed-methods systematic review “**

1 **Synthesize the findings of multiple research studies on this topic, using a rigorous and transparent methodology .Include more studies on this work.**
2 **Avoid using we, I etc in paragraphs eg:-“** **We will search for peer-reviewed articles in PubMed”,** **Systematic Reviews and MetaAnalyses guidelines will be followed ,** “**Risk of bias will be” etc……**
3 **You have generalised the Built Environment studies and physiological indicators, elaborate the work on each environment given in literature and cite the reference.**
4 **I find more redundancy in the manuscript.**
5 **You have just specified the name of biomarkers used in the study , Need to explain how it is used , outcome etc and should have complete information about each biomarker.**
6 **Table 3 is repeated content from the paragraph. What the reader can infer from the table??**
7 **Which author extract the data? Are you reviewing a work done in literature study “Data extraction will be conducted by one author (BW)”-Page 11.**
8 **Study Selection:- Need to know how to synthesize the study not how you use reference manager.**
9 **Expand the abbreviation at the beginning of the passage followed by its short form in rest of the paragraph.**
